# Longitudinal changes in cardiopulmonary outcomes of adults born extremely prematurely: United Kingdom Oscillation Study

**DOI:** 10.1038/s41390-025-04190-y

**Published:** 2025-06-16

**Authors:** Allan Jenkinson, Christopher Harris, Mona Bafadhel, Reza Razavi, Theodore Dassios, Anne Greenough

**Affiliations:** 1 Department of Women and Children’s Health, School of Life Course and Population Sciences, Faculty of Life Sciences and Medicine, King’s College London, London, UK; 2 Neonatal Intensive Care Unit, King’s College Hospital NHS Foundation Trust, London, UK; 3 King’s Centre of Lung Health, School of Immunology and Microbial Sciences, Faculty of Life Sciences and Medicine, King’s College London, London, UK; 4 Department of Cardiovascular Imaging, School of Biomedical Engineering & Imaging Sciences, Faculty of Life Sciences and Medicine, King’s College London, London, UK

## Abstract

**Background:**

During puberty, lung function of individuals born extremely prematurely can deteriorate putting them at risk of early chronic obstructive pulmonary disease (COPD). We hypothesise that young adults exposed to postnatal corticosteroids will have poorer lung and cardiac function, higher pulmonary artery pressures and poorer exercise tolerance compared to preterm born adults not exposed to postnatal steroids and term born adults. We further hypothesise lung function differences may be demonstrated depending on mode of ventilation at birth (high frequency oscillatory or conventional ventilation) in preterm born adults.

**Methods:**

A prospective study of participants (aged 24-28) from the United Kingdom Oscillation Study (UKOS) and term born controls. Assessments will involve comprehensive lung function, cardiac ultrasound, exercise assessments, inflammatory cell and biomarker profiling and airway microbiome assessment. The primary outcome is the ratio of forced expiratory volume in 1 s/forced vital capacity (FEV_1_/FVC); to detect a significant difference we will recruit 150 individuals. Statistical analysis will involve mixed effect models with adjustment for imbalances and sensitivity analysis.

**Discussion:**

The results may identify adults born extremely preterm at increased risk of COPD and pulmonary hypertension (PH) who might benefit from interventions to delay the onset of COPD and cardiovascular complications such as PH.

**Impact:**

- Adults born extremely prematurely in the modern era of neonatal care are an emerging population whose long-term outcomes have infrequently been reported.
- This study will describe their cardiac and lung function, pulmonary artery pressures, exercise capacity and immunobiological profile.
- We aim to identify risk factors for worse outcomes such as early chronic obstructive pulmonary disease onset and pulmonary hypertension.
- The results will identify those who might benefit from multi-disciplinary follow-up to ensure interventions are employed to delay the onset of COPD and manage longer term cardiovascular problems.

## Introduction

Extremely prematurely born infants (<28 weeks gestational age) make up 6% of all preterm admissions to the neonatal unit.^1^ Extremely premature infants can develop significant cardiopulmonary morbidities including chronic lung disease (bronchopulmonary dysplasia [BPD]), pulmonary hypertension (PH), morphological changes in heart structure and reduced exercise tolerance. As a consequence, an estimate of the incremental cost for an extremely preterm child surviving to 18 years is £94,740 as compared to £22,885 for a term survivor.^2^

Chronic respiratory morbidity is more common in infants who had BPD. They have more hospital admissions, more frequent respiratory symptoms requiring treatment and lung function abnormalities that persist into childhood and adolescence compared to those who did not have BPD.^3,4^ Chronic respiratory morbidity, however, can also occur in infants who did not develop BPD. Indeed, in a meta-analysis of the results of 55 cohorts, there was lower forced expiratory volume in 1 s/forced vital capacity (FEV_1_/FVC) in those born prematurely compared to those born at term, irrespective of a history of BPD.^5^

Puberty is the last positive effector of lung function with rapid growth of the airways and lung parenchyma.^6^ Yet studies have demonstrated that prematurely born adolescents may not achieve the same level of lung function during puberty as their term born counterparts.^7^ Indeed, lung function may deteriorate during puberty in those who had BPD,^8,9^ particularly if they received corticosteroids postnatally.^10^ Corticosteroids are administered to prematurely born infants to facilitate early extubation and reduce the incidence of BPD. In the United Kingdom Oscillatory Study (UKOS) cohort, postnatal corticosteroid exposure was shown to be associated with impaired lung function at 11–14 years^10^ and 16–19 years.^11^ Indeed, during that time period the lung function of those exposed to corticosteroids had declined, putting them at risk of the early development of chronic obstructive pulmonary disease (COPD).^10,11^ Given the evidence for chronic respiratory morbidity in childhood and adolescence, whether extremely prematurely born adults who were cared for in the modern era of neonatal intensive care (routinely administered antenatal corticosteroids and postnatal surfactant) are at increased risk of further respiratory morbidities and the premature onset of COPD needs urgently testing.

Despite the well reported risks of corticosteroids, our recent survey demonstrated that 100% of tertiary units are still prescribing systemically administered corticosteroids.^12^ It is therefore important to determine if lung function in adulthood has deteriorated further in those exposed to corticosteroids in the neonatal period as compared to those unexposed. This would emphasise that prescribing practices and administration of postnatal corticosteroids should be reviewed.

Preterm infants have altered immune regulation and are at risk of sustained inflammation.^13^ They are also at risk of dysbiosis, an alteration in the composition or diversity of their microbiome, which can result in invasion of virulent species and immune disruptions.^14^ The potential link between airway dysbiosis, sustained inflammation and long-term outcome has not been evaluated in extremely prematurely born adults^14^ and is one of our aims.

BPD is often associated with pulmonary vascular disease and secondary PH. A case control study from the Swedish pulmonary arterial hypertension registry demonstrated that premature birth was associated with a three-fold increased risk for adult PH.^15^ We showed in the UKOS cohort, nearly half of 193 adolescents with a mean gestational age at birth of 27 weeks had a mean pulmonary artery pressure (PAP) greater than 20 mmHg when aged 11 to 14 years of age.^16^ The presence of PH and its contribution to worse lung and heart function requires investigation, especially in the context of growing evidence of abnormal cardiac morphology in individuals born prematurely.

Individuals born preterm have been shown to have unique cardiac phenotypes with altered morphology and functional capacity in childhood and adolescence with evidence of increased risk of early cardiovascular disease.^17,18^ The UKOS cohort offers a unique opportunity to characterise these unique cardiac features in a large number of adults born extremely prematurely in the modern era of neonatal care.

Lung and cardiac function abnormalities seen in extremely prematurely born young people may translate into poorer exercise capacity. In one study^19^ young people born before 29 weeks of gestation with an abnormal forced expiratory flow at 75% of the forced vital capacity (FEF_75_) had a reduced sprint distance of 114 m and those with a very abnormal FEF_75_ had a reduced sprint distance of 159 m. Putting those figures in context, a cardiac rehabilitation programme in adults improved the results of a modified shuttle sprint test by an increase of 70 m.^20^ Furthermore, in preterm adults aged between 18 to 26 years, a decline in exercise capacity was noted for each week of gestational age.^21^ Leisure time physical exercise was lower at 18 years in those born preterm.^22^ In a study of 228 very low birth weight adults and 100 controls aged between 26 and 30 years, reduced exercise capacity was explained by impaired lung function, altered left ventricular structure and function and reduced physical exercise.^23^ We aim to investigate whether this is true in extremely prematurely born adults born in the modern era of neonatal intensive care to identify individuals who might benefit from rehabilitation programmes.

Hypothesis

- Young adults born extremely prematurely and exposed to postnatal corticosteroids will have poorer lung and cardiac function, higher pulmonary artery pressures and poorer exercise tolerance compared to young adults born extremely prematurely and not exposed to postnatal corticosteroids.
- Young adults born extremely prematurely will have worse exercise capacity compared to term born infants, and this may be explained by their abnormal respiratory and cardiac function and exercise habits.
- Lung function results will differ between young adults born extremely prematurely who received high frequency oscillatory ventilation or conventional ventilation from birth recruited into the randomised UKOS study.

## Methods

### Study design

This is a prospective cohort study of adults (aged 24–28 years) born prematurely (before 29 weeks gestation) and term born controls. The study duration will be 24 months, with a one month set up period, 18 months of assessments and five months of analysis and write up. The study aims to recruit 200 individuals: 150 participants from the UKOS cohort and 50 control term born participants.

### Setting

Assessments will be performed in King’s College London research facilities at King’s College NHS Foundation Trust Hospital.

### Eligibility criteria

Inclusion criteria are as follows:

- Young people who are now 24–28 years old who were born prematurely (below 29 weeks of gestation) and recruited into the UKOS study and term born controls.

Exclusion criteria

- Participants who are pregnant.

### Recruitment

Participants with a history of premature birth (<29 weeks gestational age) will be recruited from the UKOS database. UKOS participants have consented to receive yearly birthday cards, season greetings cards and information with regards to upcoming studies. We have communicated the current study proposal via email. Those who have indicated a positive response to participate in the research study will be further contacted by email/post/telephone to share further study information with formal invitations to be sent by email. Where there is no response to the initial e-mail correspondence, a second email will be sent or where we have contact information we will attempt to contact participants by phone.

Term participants will be recruited as controls by local advertising.

### Consent

Consent will be taken when the participants attend for cardiopulmonary tests by the research fellow who is up to date with Good Clinical Practice training and has a comprehensive understanding of the research project and the assessments involved. Consent will be in the form of a written declaration, with the option for participants to withdraw from the study at any time should they choose. Participants will be given the opportunity to ask any questions they may have at the time of consent.

### Assessment

Participants will complete questionnaires on entry to the study. These will cover questions related to their demographics (Table 1), school performance, level of education, employment history, social history and exercise habits (Supplementary material: Questionnaire), their quality of life (World Health Organization Quality of Life Brief Version [WHOQOL-BREF]); their cognition (Montreal cognitive assessment tool [MoCA]); as well an assessment of their mood with anxiety and depression scores (Patient Health Questionnaire—9 [PHQ-9] and the Generalised Anxiety Disorder Questionnaire– 7 [GAD-7]).

**Table 1:** Demographics data collection sheet

Demographics
**Maternal**
Race (white/black/other)
Smoking during pregnancy (yes/no)
Antenatal steroids (yes/no; If yes: complete course/incomplete course; number of courses)
**Neonatal**
Male sex
Birthweight (g)
Birthweight (z score)
Gestation at birth (weeks)
Multiple birth (Yes/No; if yes: twin/triplet/quadruplet)
Surfactant administration (yes/No; if yes: number of doses)
Mode of ventilation at birth (Conventional ventilation/High frequency ventilation)
Postnatal glucocorticoids (yes/No; if yes: number of courses)
Oxygen dependence at 36 weeks of post menstrual age (yes/no)
Oxygen dependency at 28 days (yes/no)
Oxygen dependent at discharge (yes/no)
**Follow up at 24–28**
Age (year)
Weight (kg)
Height (cm)
Cotinine level (ng per mL)
Smoker (yes/no; if yes: cigarettes/Vapes [yes/no]; if no: lives with smoker [yes/no])

On the day of testing, they will have a comprehensive suite of lung function assessments, exercise testing, echocardiography and blood, nasal, sputum and saliva sampling.

All lung function assessments (Table 2) will be performed according to guidelines from the American Thoracic Society and the European Respiratory Society.^24^

**Table 2:** Lung function parameters data collection sheet

Lung Function Parameters
FEF_75_ z-score
FEF_50_ z-score
FEF_25_ z-score
FEF_25-75_ z-score
FEV_1_ z-score
FVC z-score
FEV_1_/FVC z-score
PEF z-score
DLCO z-score
TLC_pleth_ z-score
FRC_pleth_ z-score
FRC_He_ z-score
RV_pleth_ z-score
R5_Hz_ z-score
R20_Hz_ z-score
LCI
FeNO ppb

*DLCO* diffusion capacity for carbon monoxide, *FeNO* fractionally exhaled Nitric Oxide, *FEF* forced expiratory flow, *FEV*_1_ forced expiratory volume in 1 s, *He* helium, *Hz* hertz, *FRC* functional residual capacity, *FVC* forced vital capacity, *LCI* lung clearance index, *PEF* peak expiratory flow, *Pleth* plethysmography, *ppb* parts per billion, *R* resistance, *RV* residual volume, *TLC* total lung capacity.

Airway function will be assessed by means of spirometric measurement of the forced expiratory flow at 75%, 50% and 25% of expired vital capacity (FEF_75_, FEF_50_, and FEF_25_, respectively), FEV_1_, and peak expiratory flow (PEF).

Airway hyperreactivity will be assessed by a bronchial challenge tailored to the baseline lung function.^25^ Those with FEV_1_  ≤  70% of predicted will receive a bronchodilator challenge and those with FEV_1_ >  70% of that predicted will undergo a bronchoprovocation challenge.^25^

Impulse oscillometry will be used to assess respiratory-system resistance.^26^ Inhomogeneity of ventilation distribution will be assessed by means of a multiple-breath technique assessing the lung-clearance index (LCI).^27,28^

Lung volumes will be assessed by means of measurements of functional residual capacity with the use of a helium-dilution technique (FRC_He_) and FVC by means of spirometry.^29^The following assessments of lung volumes will also be undertaken: functional residual capacity as assessed by means of plethysmography (FRC_pleth_) and plethysmographic assessments of total lung capacity and residual volume. Two measurements within 5% of each other will be averaged to calculate the results.^29^

The diffusing capacity of the lung for carbon monoxide (DL_CO_), alveolar volume, and gas transfer per unit volume will be assessed with the use of the single-breath gas-transfer technique.^30,31^

The fraction of exhaled nitric oxide (Fe_NO_) will be measured by means of a real-time method with the use of a computerised system and visual display. As a measure of respiratory muscle function, we will measure the maximum inspiratory and expiratory pressures using a respiratory pressure metre.

All lung-function results will be reported using established reference ranges and will be converted into z scores as appropriate.^32–37^

Participants will perform a cardiopulmonary exercise test (CPET) on a cycle ergometer to determine the maximum oxygen uptake (VO_2_) as a measure of maximum exercise capacity (Table 3). In addition, they will undergo a shuttle sprint test so that the results can be compared with those obtained when the participants were 16 to 19 years.^19^

**Table 3:** Cardiopulmonary exercise test data collection sheet

Cardiopulmonary exercise testing parameters
Peak V̇O_2_ Absolute (mL/min)
Peak V̇O_2_ Specific (mL/kg/min)
Peak V̇O_2_ (%Pred)
Peak HR (bpm)
Peak HR (%pred)
V̇E/V̇CO_2_ Slope
Peak O_2_ pulse (%pred)
Peak O_2_ pulse (V̇O_2_/HR)
Sat. O_2_ Pre
Sat. O_2_ Post
Peak V̇E (L/min)
BR (L)
BR (%)

*BPM* beats per minute, *BR* Breathing reserve, *HR* heart rate, *L* litre, *mL/kg/min* millilitre per kilogram per minute, *V̇O*_2_ Maximum rate of oxygen consumption, *Sat* Saturation, *V̇CO*_2_ maximum rate of carbon dioxide production, *V̇E* minute ventilation, *%Pred* percentage predicted.

Doppler echocardiographic studies will be undertaken to assess cardiac structure, function, PAP and diagnose PH^38^ (Table 4).

**Table 4:** Echocardiography data collection sheet

Echocardiographic measurements
TR peak velocity (m/s)
PA diameter (mm)
RVOT acceleration (time,m/s)
IVC diameter (mm)
Estimated RAP (mmHg)
SPAP (mmHg)
MPAP (mmHg)
RA area (cm^2^)
RV/LV (basal diameter ratio)
LVEDD (mm)
LVEF (%)
PI end diast. v (m/s)
PV v max (m/s)
TAPSE (cm)

*IVC* inferior vena cava, *LA* left atrium, *LV* left ventricle, *LVEDD* left ventricle end-diastolic diameter, *LVEF* LV ejection fraction, *MPAP* mean pulmonary artery pressure, *PA* pulmonary artery, *mm* milimeter, *mmHg* milimeters of mercury, *m/s* metres per second, *PAAT* pulmonary artery acceleration time, *PI* *end* *diast*. *v* end-diastolic velocity of the pulmonary regurgitation, *PV* *v max* pulmonary maximum outflow velocity, *RA* right atrium, *RAP* right atrial pressure, *RV* right ventricle, *RVOT* right ventricular outflow track, *SPAP* systolic pulmonary artery pressure, *TAPSE* tricuspid annular plane systolic excursion, *TR* tricuspid regurgitation.

Salivary samples will be obtained to assess exposure to tobacco smoke using cotinine assay. Sputum samples (spontaneous and induced) and nasal samples (nasosorption nasal brushing and nasopharyngeal swabs) will be assessed for cell count and differential to assess airway inflammation and whether there is a predominant cell type (neutrophil or eosinophil). Sputum and nasal samples will also be used to assess airway microbiome using 16 s sequencing. High-throughput 16 s sequencing will be combined with contemporaneous data collection, to assess within-subject changes in microbiome development. Blood (serum/plasma) tests will include cell count, differential and c-reactive protein (CRP). In addition we will assess circulating biomarkers associated with lung function abnormalities (CC-16, s-RAGE),^39^ neutrophilic airway inflammation (CXCL8/IL8)^40^ and a panel of cytokines associated with both t-helper 1 (Th1) and t-helper 2 (Th2) immune response which mediates the inflammatory response thus assessing the immunological profile of participants^41^ (ProcartaPlex Human Th1/Th2 Cytokine Panel: GM-CSF, IFN gamma, IL-1 beta, IL-2, IL-4, IL-5, IL-6, IL-12p70, IL-13, IL-18, TNF alpha).

### Outcome measures

This is a long-term follow up of lung function and cardiac outcomes in a group of extremely prematurely born adults who have had exposure to antenatal steroids and postnatal surfactant.

Our primary outcome is FEV_1_/FVC in extremely prematurely born adults with postnatal corticosteroid exposure versus no postnatal corticosteroid exposure.

Our secondary outcomes will include spirometric lung function, cardiopulmonary exercise test of maximum oxygen uptake (VO_2_) and PAP estimation by cardiac ultrasound of UKOS participants compared to term born controls.

We will also compare these primary and secondary outcomes in participants enrolled in the UKOS trial as per their initial designation of mode of ventilation.

### Sample size

The generally accepted lung function threshold for developing symptoms in COPD is a FEV_1_/FVC ratio below 70% of the predicted value^42^ which corresponds to a z-score of −2.19. The dexamethasone exposed group of the UKOS cohort had an FEV_1_/FVC ratio z-score of −1.83 which is above the threshold, but very close to it.^11^ In contrast the corticosteroid unexposed group had an FEV_1_/FVC ratio z-score of −0.89. The cohort was assessed at 16–19 years of age and are now ~8 years older and possibly symptomatic. Calculations have been based on the FEV_1_/FVC ratio, as it has been associated with a clinical symptom threshold.^43^ To detect a clinically significant difference in FEV_1_/FVC of 0.33 between the steroid and non steroid exposed (the difference in the mean FEV_1_/FVC z-score between the results of the 11–14 year olds and 16–19 year olds in the UKOS cohort) with a SD of the FEV_1_/FVC z-score of 1.21 and with 90% power and 5% significance, we will need 64 patients per group—128 in total. We will aim to recruit 150 patients from the UKOS cohort to allow for drop out.

To detect a clinically significant difference in the mean PAP of 2.31 mmHg as previously observed between children born preterm versus children born at term when they were 11–14 years of age with a SD of the mean PAP of 2.8 mmHg^16^ and with 90% power and 0.01 significance, we will need 44 patients per group (44 controls and 44 prematurely born young people). We will aim to recruit 50 term-born controls to allow for drop out.

### Statistical analysis

For the main analysis of outcomes, we will use mixed models, with the mother or the pregnancy as the random effect to allow for clustering due to multiple births. Skewed lung-function outcome data will be log-transformed.

All study outcome analyses will be adjusted for observed baseline imbalances between groups by incorporating the unbalanced factors as fixed effects in the multifactorial model. Sensitivity analyses will be conducted to explore the robustness of adjustment for baseline imbalances between groups. Propensity score matching, used in the follow up of UKOS patients when aged 16–19,^44^ will be utilised as part of the sensitivity analysis methodology. Unadjusted and adjusted analyses will be presented to show the effects of adjustment as estimates with 95% confidence intervals. As we have a clearly predefined single primary outcome, FEV_1_/FVC, we will not adjust for multiple testing of the secondary outcomes. Neonatal baseline data will be compared for the children recruited and not recruited at age 24–28 years to determine the representativeness of the group with follow-up data. Those variables that differ between recruited versus not recruited will be further adjusted for a sensitivity analysis. Some participants will be unable to complete all tests and, so, multiple imputation using chained equations will be used to impute missing data.

## Discussion

Longitudinal follow up of children and adolescents born preterm has provided evidence of adverse cardiopulmonary outcomes^9,45^ including abnormal lung function, increased risk of asthma, COPD, PH and abnormal cardiac morphology and function with reduced exercise tolerance.^46,47^ Many of these outcomes were reported in cohorts that were born in a period prior to widespread use of surfactant and antenatal corticosteroids.

The importance of the continued follow up of the UKOS cohort as they now reach adulthood, is emphasised by the previous findings of reduced lung function and a trend towards spirometric parameters consistent with a diagnosis of early COPD when they were adolescents, in particular in individuals exposed to corticosteroids in the neonatal period.^11^ In addition, the UKOS cohort were born in the “modern” era of neonatology with more than 90% exposed to surfactant and antenatal corticosteroids.^48^ Whether there has been a further deterioration in lung function meeting spirometry or clinical criteria for early COPD, particularly in those with postnatal corticosteroid exposure needs urgent testing.

While the focus of primary and secondary outcomes is on airway abnormalities as measured by spirometry, our methodology includes other important measurements of lung function (DL_CO_, Fe_NO_, LCI) which will provide evidence of long-term pulmonary gas exchange, airway inflammation and ventilation inhomogeneity.^49^ In addition, prematurity has been shown to affect respiratory muscle function with prematurely born infants having decreased maximal expiratory pressures,^50^ but it is not known whether the effect of prematurity persists to adulthood. This study will establish the respiratory muscle reserves of prematurely born adult individuals which is important as they are thought to present with early COPD.^51^

There is evolving evidence of pulmonary hypertension as a complication of preterm birth. We have previously shown nearly half of 193 adolescents with a mean gestational age at birth of 27 weeks had a mean PAP greater than 20 mmHg when aged 11 to 14 years of age.^16^ It has recently been shown that young adults born prematurely have early pulmonary vascular disease characterised by elevated pulmonary pressures, a stiffer pulmonary vascular bed and right ventricular dysfunction consistent with an increased risk of developing PH.^52^ This study however had a cohort of 11 preterm adults without widespread surfactant and antenatal corticosteroid exposure. Exercise intolerance is a cardinal symptom of PH and strongly impacts on quality of life.^53^ Extremely prematurely born infants had reduced exercise tolerance in adolescence^19^ and this may have worsened when they are now young adults perhaps due to PH development.

Older adults born preterm are at risk of increased care episodes due to COPD, regardless of BPD status.^54^ COPD results from abnormal or persistent inflammation.^55,56^ Preterm infants have altered immune regulation and are at risk of sustained inflammation.^13^ There is emerging evidence of altered immune programming and microbiome dysbiosis in the pathophysiology of obstructive lung disease after preterm birth.^57–59^ Altered T cell maturation in preterm born individuals could be of importance when considering interventions such as immunotherapy and vaccines.^60–62^ Additionally, airway dysbiosis in preterm individuals could be targeted with probiotics; there have been promising results in animal studies.^63,64^ Hence we will be investigating this in our cohort.

## Conclusion

The proposed study will detail the lung function of adults born preterm in the modern era of neonatology. It will describe cardiac function, PAP, exercise capacity and immunobiological profile of this emerging population. The results will identify those who might benefit from interventions to delay the onset of COPD and manage longer term cardiovascular problems such as PH.

## Supplementary information

PR_Supplement_Questionnaire

## Data Availability

Data will be made available on request to the corresponding author.
