# Supplementary material for: Longitudinal changes in cardiopulmonary outcomes of adults born extremely prematurely: United Kingdom Oscillation Study
Source: Pediatr Res. 2025 Jun 16;99(1):106–11. doi: 10.1038/s41390-025-04190-y (PMC12920102; doi:10.1038/s41390-025-04190-y)
Supplement: Supplementary file 1 — PR_Supplement_Questionnaire [file 41390_2025_4190_MOESM1_ESM.pdf]

KING'S College LONDON

King's College Hospital NHS
NHS Foundation Trust

UKOS

United Kingdom Oscillation Study

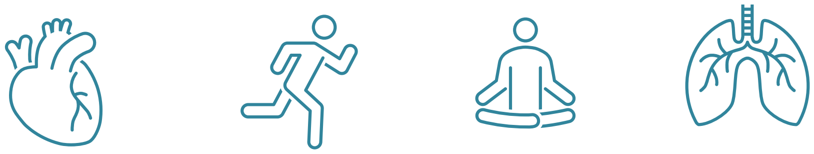

The questions in this form ask you about your usual health, how you usually feel and about your education. Please do not report temporary or occasional problems unless specified in the question.

If you have any questions, please contact:

Professor Theodore Dassios (theodore.dassios@nhs.net)

Professor Anne Greenough (anne.greenough@kcl.ac.uk)

Dr Allan Jenkinson (allan.jenkinson@kcl.ac.uk)

Neonatal Intensive Care Unit

King's College Hospital NHS Foundation Trust

4TH Floor, Golden Jubilee Wing

Denmark Hill

SE5 9RS

London

Phone Number: 020 3299 3037

Study identification number:

Date of completion:

What is your ethnic group?
Choose ONE section from A to E

A White
- ❑ British
- ❑ Irish
- ❑ Any other white background (please specify)_________________________

B Mixed
- ❑ White and Black Caribbean
- ❑ White and Black African
- ❑ White and Asian
- ❑ Any other Mixed background (please specify)_________________________

C Asian
- ❑ Indian
- ❑ Pakistani
- ❑ Bangladeshi
- ❑ Any other Asian background (please specify)_________________________

D Black or Black British
- ❑ Caribbean
- ❑ African
- ❑ Any other Black background (please specify)_________________________

E Chinese or other ethnic group
- ❑ Chinese
- ❑ Any other (please specify)____

Your Health and Well-being

1. **In the last 12 months, have you had any attacks of wheezing?**

❑ Yes ❑ No (if no, go to next question)

If yes, can you tell us approximately how frequently?

❑ Daily

❑ Weekly

❑ Monthly

❑ Less than monthly

2. **In the last 12 months, have you had wheezing when you did not have a cold?**

❑ Yes ❑ No

3. **In the last 12 months, have you taken any medications for chest problems other than antibiotics?**

❑ Yes ❑ No ❑ Don't know

If yes, can you tell us what they were from the list below?

Prednisolone		❑
Oxygen		❑
Inhalers:	Salbutamol/Ventolin	❑
	Bricanyl	❑
	Atrovent	❑
	Salmeterol	❑
	Beclomethasone	❑
	Flixitide	❑
	Combivent	❑
	Pulmicort	❑
	Seretide	❑
	Montelukast (Singulair)	❑
Others: ______________________		❑

4. In the last 12 months, have you been given any courses of antibiotics for chest problems?

❑ Yes ❑ No ❑ Don't know

If yes, can you tell us approximately how many?

5. Have you ever had asthma?

❑ Yes ❑ No

6. In the last 12 months, have you been admitted to hospital for any reason?

❑ Yes ❑ No

If yes, can you tell us the reason and number of admissions?

**Reason**	**Number of admissions**
Chest problems	
Surgery	
Mental health	
Other	

Please tell us briefly about these admissions at the back of this booklet

7. Do you smoke?

❑ Yes ❑ No

8. Does anyone you live with smoke?

❑ Yes ❑ No

9. Do you drink alcohol?

❑ Yes ❑ No

10. How many units of alcohol do you drink per week ?

11. How many hours a week do you normally exercise so much that you get out of breath or sweat?

Educational Attainment

1. **What is the highest level of education you have completed?**
   ☐ No formal qualifications
   ☐ GCSEs or equivalent
   ☐ A-Levels or equivalent
   ☐ Apprenticeship or vocational qualification (e.g., NVQ, BTEC)
   ☐ Foundation Degree or equivalent (e.g., HND, HNC)
   ☐ Bachelor's Degree (e.g., BA, BSc)
   ☐ Master's Degree (e.g., MA, MSc)
   ☐ Doctorate (e.g., PhD, DPhil)
   ☐ Other (please specify): ___________________________

2. **Did you attend university or a higher education institution?**
   ☐ Yes, I completed my degree
   ☐ Yes, but I did not complete my degree
   ☐ No, I did not attend university

3. **Are you currently studying for any educational qualifications?**
   ☐ Yes, full-time
   ☐ Yes, part-time
   ☐ No

4. **At what age did you leave full-time education?**
   ☐ 16 or younger
   ☐ 17-18
   ☐ 19-21
   ☐ 22 or older

Employment Status

5. **What is your current employment status?**
☐ Employed full-time (35+ hours per week)
☐ Employed part-time (less than 35 hours per week)
☐ Self-employed
☐ Unemployed and looking for work
☐ Not in employment and not looking for work
☐ In full-time education or training
☐ In part-time education or training
☐ Other (please specify): ____________________________

6. **Which of the following best describes your current role or occupation?**
☐ Professional (e.g., teacher, engineer, solicitor)
☐ Skilled trade (e.g., electrician, plumber, chef)
☐ Administrative or clerical
☐ Manual or unskilled labour
☐ Creative or arts-based
☐ Student
☐ Not applicable
☐ Other (please specify): ____________________________

7. **Have you been employed at any point in the last 12 months?**
☐ Yes, continuously employed
☐ Yes, but with gaps in employment
☐ No, I have not been employed

8. **What is your current job sector? (if applicable)**
☐ Healthcare
☐ Education
☐ Technology/IT
☐ Finance/Banking
☐ Hospitality/Leisure
☐ Retail/Customer Service
☐ Manufacturing/Construction
☐ Public Sector/Government
☐ Other (please specify): ____________________________
☐ Not applicable

9. **Are you satisfied with your current employment status?**
   ☐ Very satisfied
   ☐ Somewhat satisfied
   ☐ Neutral
   ☐ Somewhat dissatisfied
   ☐ Very dissatisfied

10. **If you are unemployed, what are the main reasons? (Tick all that apply)**
   ☐ Lack of available jobs in my area
   ☐ Lack of qualifications or experience
   ☐ Health issues or disability
   ☐ Caring responsibilities
   ☐ Currently pursuing education or training
   ☐ Other (please specify): ______________________________
   ☐ Not applicable
